# Structural and Dynamic Features of F-recruitment Site Driven Substrate Phosphorylation by ERK2

**DOI:** 10.1038/srep11127

**Published:** 2015-06-08

**Authors:** Andrea Piserchio, Venkatesh Ramakrishan, Hsin Wang, Tamer S. Kaoud, Boris Arshava, Kaushik Dutta, Kevin N. Dalby, Ranajeet Ghose

**Affiliations:** 1 Department of Chemistry, The City College of New York, New York, NY 10031; 2 Division of Medicinal Chemistry, University of Texas, Austin, TX 78712; 3 Department of Medicinal Chemistry, Faculty of Pharmacy, Minia University, El-Minia 61519, Egypt; 4 Department of Chemistry, The College of Staten Island, Staten Island, NY 10314; 5 The New York Structural Biology Center, New York, NY 10027; 6 Graduate Center of CUNY, New York, NY 10016.

## Abstract

The F-recruitment site (FRS) of active ERK2 binds F-site (Phe-x-Phe-Pro) sequences found downstream of the Ser/Thr phospho-acceptor on cellular substrates. Here we apply NMR methods to analyze the interaction between active ERK2 (ppERK2), and a 13-residue F-site-bearing peptide substrate derived from its cellular target, the transcription factor Elk-1. Our results provide detailed insight into previously elusive structural and dynamic features of FRS/F-site interactions and FRS-driven substrate phosphorylation. We show that substrate F-site engagement significantly quenches slow dynamics involving the ppERK2 activation-loop and the FRS. We also demonstrate that the F-site phenylalanines make critical contacts with ppERK2, in contrast to the proline whose *cis-trans* isomerization has no significant effect on F-site recognition by the kinase FRS. Our results support a mechanism where phosphorylation of the disordered N-terminal phospho-acceptor is facilitated by its increased productive encounters with the ppERK2 active site due to docking of the proximal F-site at the kinase FRS.

The mitogen activated protein kinase (MAPK) ERK2, a serine/threonine kinase, is activated downstream of the Ras-Raf-MEK cascade in response to a variety of extracellular cues including growth factors, cytokines, hormones, neurotransmitters and osmotic stress^1^,^2^. ERK2 contains a canonical two-lobed catalytic domain characteristic of eukaryotic kinases. In lieu of external regulatory domains, it contains a 30-residue insertion (MAP kinase insert) between helices αG and αH in the catalytic domain as well as an extended C-terminus^3^. Activation occurs upon dual-phosphorylation on an activation-loop TxY (x is any amino acid) motif. Active ERK2 (ppERK2) is capable of phosphorylating a large array of substrates located in the cytosol, in various cellular organelles and in the nucleus where ERK2 controls critical cellular processes including transcriptional activation^4^,^5^,^6^, chromatin remodeling^7^ and nuclear translocation^8^.

ERK2 mediated phosphorylation occurs on a consensus Px(S/T)P sequence, though only the (S/T)P motif appears to be strictly necessary^9^,^10^. In spite of its functional necessity, this motif alone is not sufficient to impart sufficient affinity or selectivity towards substrates^10^. In fact, ERK2 and other MAP kinases utilize two modular docking sites^11^ located outside the active site of the enzyme to selectively direct themselves to specific cellular targets. The first, termed the D-recruitment site (DRS)^12^,^13^, contains two contiguous sub-sites: the Φ_hyd_ sub-site located behind the ATP binding site provides mostly hydrophobic contacts; the Φ_chg_ sub-site^14^, confined within a single loop, provides electrostatic stabilization. The DRS selects substrates that contain a so-called D-site (also called the D-domain or the DEJL motif) sequence, R/KxxR/Kx_1–6_LxL^15^, which is located downstream of the phosphorylation motif. A host of structural techniques including crystallography and NMR^16^ have revealed the interactions between D-site sequences and the DRS. A second docking site, the F-recruitment site (FRS), is situated on a surface opposite the DRS below the activation-loop. The FRS is fully formed only upon ERK2 activation^17^ and targets substrates carrying an F-site (also known as a DEF motif) consensus sequence (FxFP), with phosphorylation sites located at variable distances from its N-terminus^18^,^19^. F-sites are found in a variety of ERK2 substrates, most notably the transcription factors of the Ets-family, including Elk-1, Lin-1^18^ and Sap-1^20^. In Elk-1, which contains both a D-site and an F-site, F-site/FRS interactions have been demonstrated to drive phosphorylation to specific sites^19^,^21^ that are crucial for DNA binding and transcriptional activation^22^. While hydrogen exchange mass spectroscopy (HXMS) combined with site-directed mutagenesis has helped identify key ERK2 residues that form the FRS^17^, very little specific insight is available on the structural and dynamic characteristics of F-site sequences, their interactions with the FRS, and FRS-driven substrate phosphorylation. Using solution NMR techniques we investigated the interactions of ppERK2 with a 13-residue peptide derived from Elk-1 (*Elk*_*387-399*_) that encompasses a phosphorylation site (S389) and an F-site (**F395**-Q396-**F397**-**P398**). These studies represent the first detailed analysis of the structural and dynamical characteristics of FRS/F-site docking and its role in F-site driven phosphorylation. Our results suggest that while the two phenylalanine residues (of the FxFP sequence) make significant stabilizing contacts with ppERK2 and appear to be critical for docking interactions, the terminal proline is less important. We also find that the phosphorylation motif and the F-site are uncoupled structurally and dynamically and while the former is disordered, making few contacts with ppERK2, it is nevertheless phosphorylated. This provides support for the mechanism of proximity-mediated catalysis that proposes that docking interactions serve to increase the effective local concentration of the substrate moiety, thereby enhancing its probability of productively encountering the ppERK2 active site resulting in phosphorylation^23^.

## Results

### *Elk*_387-399_ reduces conformational dynamics in ppERK2

We previously reported the partial backbone assignment of inactive ERK2^14^,^24^; ppERK2, in contrast, exhibits behavior (rapid transverse relaxation, tendency to aggregate at high concentration etc.) not conducive to standard backbone-directed resonance assignment strategies. We noticed, however, that the presence of a five- to ten-fold excess of *Elk*_*387-399*_ results in significantly reduced relaxation rates that remain constant for concentrations upto ~200 μM, and produce spectra with significantly improved dispersion. Employing previously described methodology^24^ we were able to assign 213 ^15^N, ^1^H resonances (63% of 338 amides expected; ~260 resonances observed at 800 MHz) of ppERK2 bound to *Elk*_*387-399*_. Further, upon utilizing these resonance assignments and ^15^N, ^1^H TROSY-based titrations of ppERK2 with *Elk*_*387-399*_ (and in few instances, by comparison with inactive ERK2 spectra), we could assign 182 resonances belonging to ligand-free ppERK2 (54% of 338 expected; ~213 observed). Several resonances corresponding to residues in the MAPK insert (helices α1L14, α2L14 and the distal αL16), on helix αG, on the activation-loop, the P+1 loop (located at the back end of the activation loop and involved in the docking of the critical proline residue on the substrate^1^,^2^), and on the loop hosting the HRD motif (a conserved motif involved in the coordination of the substrate hydroxyl group and in shaping the configuration of the overall catalytic machinery)^1^,^2^ are severely broadened (to below the noise threshold) in free ppERK2, but re-appear in the presence of *Elk*_*387-399*_, suggesting significant quenching of dynamics in the μs-ms timescale (Supplementary Fig. S1). Chemical shift perturbations (CSP) observed for the critical Y261^17^ (rat ERK2 numbering used throughout) and for resonances belonging to helix αG indicate that, as expected, the peptide engages the FRS (Supplementary Fig. S1). However CSPs alone do not allow a complete mapping of the peptide-binding site, since a number of ppERK2 resonances for residues in this region are not visible, especially in the absence of *Elk*_*387-399*_.

### The FRS and DRS on ppERK2 are not allosterically coupled

**Figure 1:**
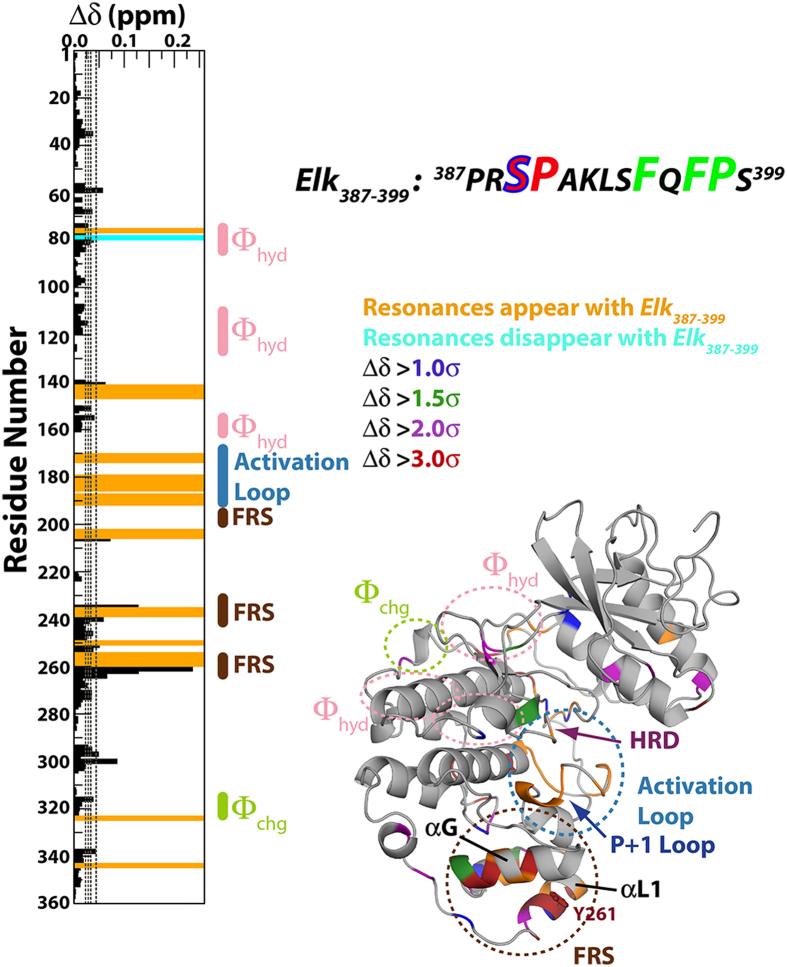
Spectral perturbations induced by *Elk*_*387-399*_ (kinase-to-peptide ratio 1:2.8) on ppERK2 in the presence of a 20-fold excess of *KIM*_*15-31*_ with the *KIM*_*15-31*_-saturated state taken as reference. Perturbations plotted against residue number are shown on the left panel. The right panel displays the perturbations mapped onto the structure of ppERK2. Δδ values that are 1.0σ, 1.5σ, 2.0σ, 3.0σ above the mean are colored blue, green, purple and red respectively. The position of the critical Y261 on the FRS is indicated. The FRS (brown), the activation-loop (blue) and the Φ_chg_ (lime-green) and Φ_hyd_ (pink) components of the DRS are circled. The HRD and the P+1 motifs are indicated by blue and magenta arrows, respectively. Residues that appear or disappear in the presence of *Elk*_*387-399*_ are colored orange and cyan, respectively for both the left and right panels. Also shown is the sequence of *Elk*_*387-399*_ with the phosphorylation (SP) motif (the phosphorylatable S389’ shown with a blue outline) and the F-site (FxFP) sequence shown in larger font and colored red and green respectively.

An unexpected finding of our NMR-based titration experiments was the observation that *Elk*_*387-399*_also appears to engage the DRS at high concentration. All the key components of the Φ_hyd_ and Φ_chg_ DRS sub-sites are extensively perturbed (Supplementary Fig. S1), including Loop 4 (N80, G83), helix αD (L113), Loop 8 (T116-S120), helix αE (D122-Y126), Loop 11 (L155-N156, T158-C159), Loop 16 (Y314-D316, D319) and helix αI (E303, Q304). As further evidence that *Elk*_*387-399*_does indeed engage the DRS, we noted a small but significant increase in the K_M_ (10.1 ± 0.8 μM to 22.7 ± 2.8 μM) for the phosphorylation of a high-efficiency D-site containing substrate (ERK-sensor-D1, see Methods) in the presence of *Elk*_*387-399*_ (100 μM) without any concurrent changes in the V_max_ (21.4 ± 0.5 and 23.4 ± 1.1 nM phosphate/second, Supplementary Fig. S2). In order to confirm that the perturbations at the DRS reflect direct binding rather than some degree of allosteric coupling between the FRS and the DRS, we tested the ability of *Elk*_*387-399*_to bind inactive ERK2. It is to be noted that the FRS is only formed fully in ERK2 upon activation^17^. A five to one excess of *Elk*_*387-399*_does not result in any significant perturbations at the FRS or in the activation-loop of inactive ERK2, but perturbations, similar to those seen in the case of ppERK2, are seen at the DRS and the constituent Φ_hyd_ and Φ_chg_ sub-sites (Supplementary Fig. S1). As a further control, we titrated ppERK2 with a DRS specific ligand (*KIM*_*15-31*_, K_D_ = 1.3 μM); as previously reported for inactive ERK2^25^, the only significant CSPs are seen at the DRS and no significant long-range perturbations including at the FRS, are noted (Supplementary Fig. S1). Finally, we repeated the titration of ppERK2 with *Elk*_*387-399*_in the presence of a saturating amount *KIM*_*15-31*_. In this case, the spectral perturbations seen at the FRS are reproduced while the residues at the DRS do not show any further perturbations in the presence of increasing amounts of *Elk*_*387-399*_ (Fig. 1). Thus as suggested by our previous kinetic studies on modular ERK2 substrates, the DRS and FRS do not exhibit any direct allosteric coupling^26^.

The interaction between *Elk*_*387-399*_ and ppERK2 may therefore be formally described as involving binding to two independent sites (the FRS and the DRS) of unequal affinity. Due to the large difference between the K_D,FRS_ and the K_D,DRS_ (see below), we were able to obtain an excellent estimate of the K_D,FRS_ using isothermal titration calorimetry (ITC) that employed lower concentrations of ppERK2 and *Elk*_*387-399*_ than used for the NMR experiments (Supplementary Fig. S3a). Fitting the ITC data to a single-site model (see justification below) produces a K_D,FRS_ of 8.0 ± 0.9 μM. This ITC-determined value was fixed and used to globally fit the CSPs for specific DRS residues to a two-site exchange model (Supplementary Fig. S3b) to obtain a K_D,DRS_ of 242.0 ± 15.5 μM (note for the ITC data, the occupancy of the DRS is ~6% at an equimolar ppERK2:*Elk*_*387-399*_ ratio at the concentrations employed).

### Spectral perturbations induced by ppERK2 on *Elk*_387-399_ suggest localized interactions between the FRS and the F-site

**Figure 2:**
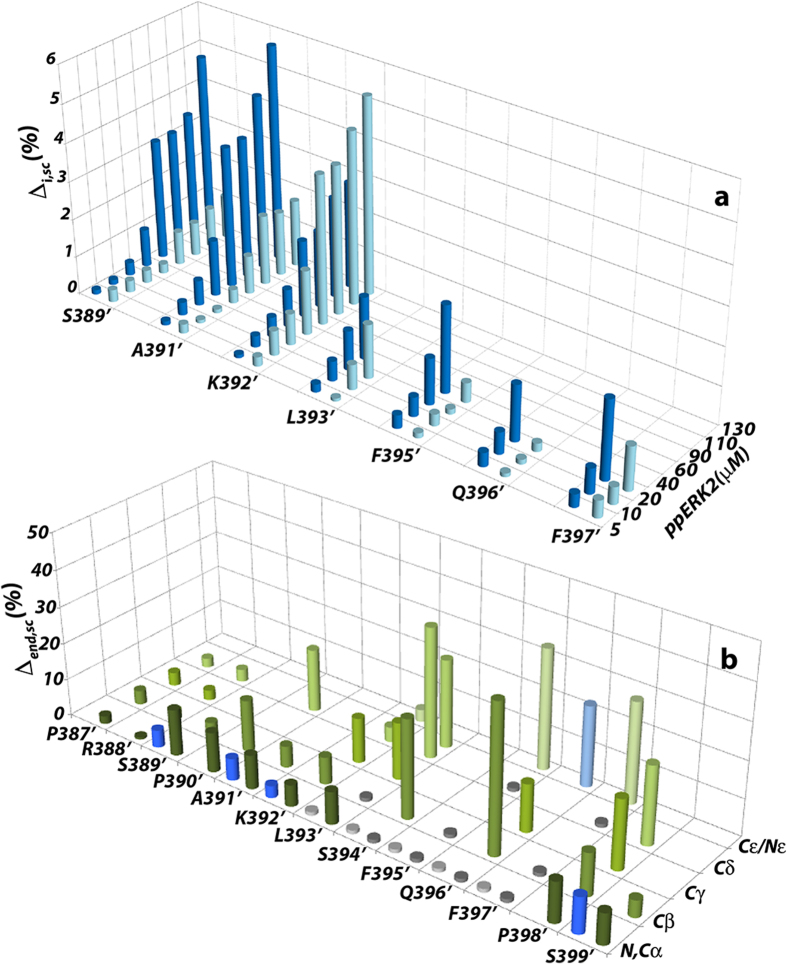
Spectral perturbations induced by ppERK2 on *Elk*_*387-399*_. (**a**) Chemical shift perturbations (Δ_i,sc_) at various stages of the titration shown for amides exhibiting both resonances belonging to *trans*-containing (dark-blue) and *cis-*containing (light-blue) conformers. (**b**) Chemical shift perturbations (Δ_end,sc_) at the titration end-point for nitrogen-containing (blue) or carbon-containing positions (green). The colors of the bars become progressively lighter with the position along the length of the sidechain. Light grey and dark grey bars indicate backbone amide and aliphatic positions, respectively, that disappear during the course of the titration. Data were acquired at 700 MHz.

In order to further investigate the mechanism of FRS-directed substrate recognition and phosphorylation we produced *Elk*_*387-399*_ labeled with NMR-active isotopes (uniformly-^15^N, ^13^C or uniformly-^15^N, ^2^H, ^13^C-labeled) and probed its interactions with unlabeled (or ^2^H-labeled) ppERK2. The ^1^H, ^15^N HSQC (or TROSY) based titrations allowed classification of the amide resonances into two main groups suggesting a difference in their overall structural and dynamic characteristics upon docking onto ppErk2 (Fig. 2a). Amides belonging to residues encompassing the *L393’-F397’* fragment (*Elk*_*387-399*_ residues are italicized and primed) that includes the F-site (FxFP) disappear at early stages of the titration when 14-28% of *Elk*_*387-399*_ is bound to ppERK2, and do not reappear even when ~97% of uniformly ^2^H, ^15^N-labeled *Elk*_*387-399*_ is bound to ^2^H-labeled ppERK2. The fact that protonated and deuterated samples show similar behavior suggests that the signal disappearance observed in the *L393’-F397’* segment is the result of chemical exchange on the intermediate timescale rather than efficient relaxation due to the slow molecular tumbling of the complex. These resonances were not recovered even at saturating concentrations of ppERK2, suggesting that the exchange phenomenon likely involves structural fluctuations occurring after complex formation. The remaining amides, including the phosphorylation target *S389’,* display no significant line broadening and are detectable at every stage of the titration (Fig. 2a), suggesting that they retain a high degree of flexibility in the complex. It should be noted that, given the tendency of *Elk*_*387-399*_ to also bind at the DRS, albeit at high-concentrations, the CSPs described above for uniformly-^15^N, ^13^C labeled *Elk*_*387-399*_ were obtained in the presence of ppERK2 containing saturating amounts of *KIM*_*15-31*_ to block the DRS, and reflect interactions exclusively with the FRS.

A higher level of detail on the role of specific sidechains of *Elk*_*387-399*_ in its interactions with ppERK2 was obtained from ^13^C, ^1^H HSQC-based titration acquired up to an equimolar *Elk*_*387-399*_ to ppERK2 ratio (78% bound). In order to compare the magnitudes of the perturbations for different sidechain positions (Fig. 2b), we extended the concept of residue-specific weighting factor, previously introduced by others^27^, to include sidechain atoms. Briefly, we postulate that the distribution of chemical shifts deposited in the BMRB ( http://www.bmrb.wisc.edu) for a given atom reflects the variety of chemical environments experienced by it in the entire protein fold space. For a given atom, we expressed the perturbation (Δ_sc_, Equation 2) experienced upon binding as the percent chemical shift change with respect to the standard deviation of BMRB-deposited chemical shifts for that atom. As a first approximation, when two chemically distinct moieties undergo comparable conformational changes their corresponding Δ_sc_ values are predicted to be similar. A number of sidechain resonances for the *L393’-F397’* fragment broaden beyond the threshold of detection at the same stages of the titration as seen for the backbone amide resonances (Fig. 2b). This includes all α-positions (except *L393*’ α), and the β-positions of *L393*’, *F395’, Q396’* and *F397*’. However, the peripheral positions for the long-chain amino acids (*L393’* γ, δ1,2; *S394’* β; *Q396’* δ, ε) and to a lesser extent, the aromatics, though significantly broadened, maintain sufficient mobility on the ps-ns timescale, to be detectable at an equimolar concentration of *Elk*_*387-399*_ and ppERK2 (Fig. 2b). The largest Δ_end,sc_ values (Δ_sc_ at the titration end-point) also predominantly cluster on the *L393’-F397’* fragment where the average perturbation per residue is consistently above 20%. In particular, aromatic resonances corresponding to *F395’* and *F397’* and the β-methyl resonances of *L393’* displayed Δ_end,sc_ values of approximately 30%. These represent large perturbations in absolute terms and strongly suggest direct contact between these *Elk*_*387-399*_ sidechains and ppERK2. In comparison, at the far C-terminus of *Elk*_*387-399*_, not only are resonances corresponding to all positions of *P398*’ (the last residue of the FxFP motif) visible at the end of the titration, they show significantly smaller Δ_end,sc_ values than the still observable resonances of the neighboring *L393’-F397’* fragment. The terminal *S399’* showed vanishingly small Δ_end,sc_ values. At the N-terminus around the SP phosphorylation motif, the largest average perturbation is seen for *P390’*; the phosphorylation target, *S389*’ shows similar changes at the α position, but the β position displays a very small but detectable Δ_end,sc_. The first two residues at the N-terminus of *Elk*_*387-399*_ do not display any significant Δ_end,sc._ Importantly, the addition of MgCl_2_ followed by a saturating amount of AMPPCP (which contains a non-transferable γ phosphate) does not significantly affect *S389*’ or any other resonances. Yet, incubation of *Elk*_*387-399*_ with ATP/Mg^2+^ and ppERK2 converts *S389’* entirely into phospho-serine (enzymatic assays suggest a k_cat_=0.56 ± 0.1 sec^−1^; K_M_=13.4 ± 1.1 μM; Supplementary Fig. S4a), as indicated by the characteristic large downfield chemical shift changes observed for *S389’* (for the amide resonance and for that corresponding to the β position, Supplementary Figs. S4b,c)^28^. This confirms that *Elk*_*387-399*_ is a viable substrate for ppERK2 under the conditions employed for our NMR experiments.

**Figure 3:**
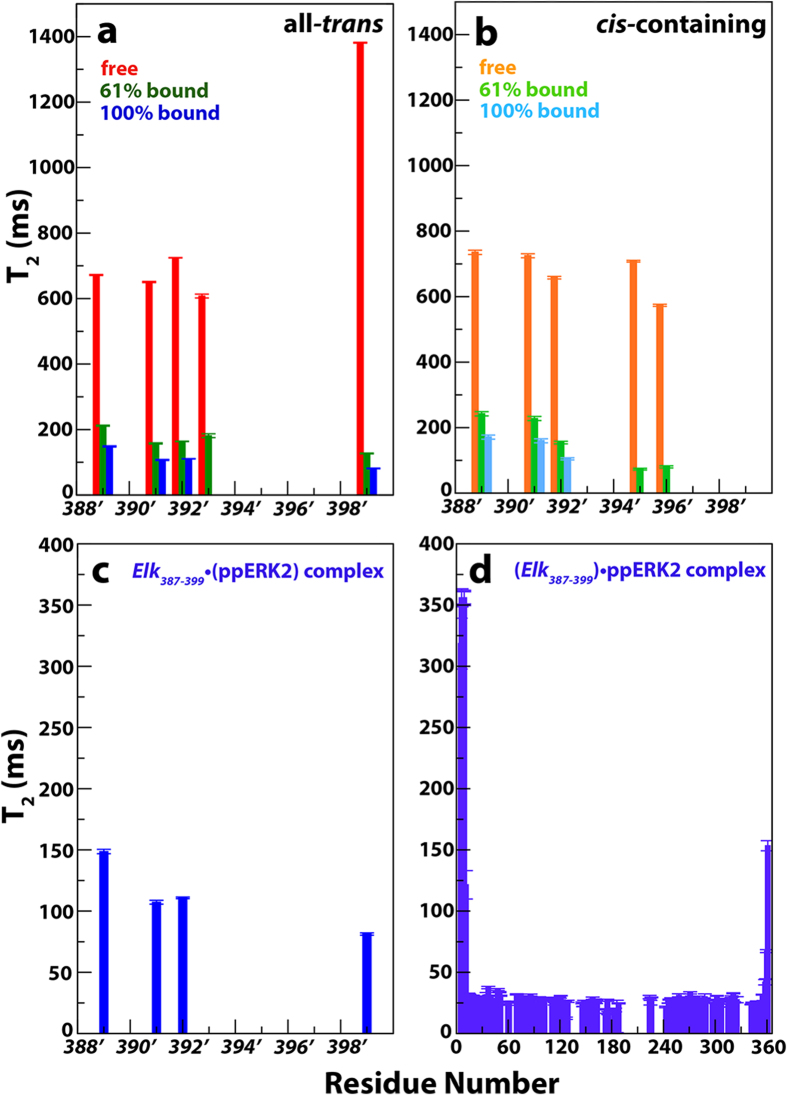
Backbone ^15^N transverse relaxation times (T_2_) for the (**a**) major all-*trans* and (**b**) minor *cis*-containing components of *Elk*_*387-399*_. Data is shown only for those resonances that are observable in the state that is 61% bound to ppERK2 (assuming a K_D_ = 8 μM). T_2_s for the 100% bound state are extrapolated from those of the free and 61% bound states assuming a fast exchange on the timescale defined by the relaxation rates. Extrapolated fully bound T_2_ values are only shown for those residues (*S389’*, *A391’*, *K392’* and *S399’*) that display minimal line broadening during titration course and are in the fast exchange regime. Note that residues *P390’* and *P398’* are prolines and resonances close to the F-site disappear early in the titration course. The *cis-*containing conformers of *F395’* and *Q396’* show T_2_ values in 61%-bound state that are significantly shorter than those of the N-terminus and approach those of the core residues of ppERK2 (shown in panel d, see below) containing saturating amounts of *Elk*_*387-399*_. This suggests that the F-site sequence in these minor states also engages the ppERK2 FRS. An expansion of the extrapolated T_2_s of the all-*trans* conformers of Elk_387*-399*_ when fully-bound to ppERK2 is shown in (**c**) on the same scale as those of ppERK2 in the presence of saturating amounts of *Elk*_*387-399*_ (**d**) to allow direct comparison. It is evident that the T_2_ values for the N-terminus of *Elk*_*387-399*_ are higher than those for the ppERK2 core but shorter than those in the extreme N- and C-termini. This suggests that while the N-terminus of *Elk*_*387-399*_ is partially constrained by its F-site that is firmly docked onto ppERK2, it is still flexible.

We were also able to measure the ^15^N spin-spin relaxation times (T_2_) at 600 MHz (Fig. 3a) for the still-visible residues of *Elk*_*387-399*_ (61% bound): *S389’* (212 ms), *A391’* (157 ms), *K392’ (*164 ms), *L393’* (181 ms) *S399*’ (127 ms) and *cis*-containing minor states (Fig. 3b) for *S389’* (242 ms), A*391’* (228 ms), *K392’* (154 ms), *F395’* (80 ms) and *Q396’* (73 ms). Assuming a fast-exchange regime such that the measured relaxation times are population-weighted averages of those in the free and bound states, then the extrapolated fully bound T_2_ values for the *S389’*-*K392’* segment were found to lie in the 107-149 ms range (Fig. 3c). These values lie between those for the amides of the rigid core of *Elk*_*387-399*_-bound ppERK2 (Fig. 3d; ~30 ms, indicative of a monomeric ppERK2 in line with previous results^29^) and its flexible N (350 ms) and C-terminal tails (150 ms) (Fig. 3d). Taken together, the spectral perturbations and relaxation data support the scenario in which the bulk of the interactions between *Elk*_*387-399*_and the ppERK2 involve the side chains of *L393’*, *F395’*, *F397’* and, to a lesser extent, *K392’*, *Q396*’ and *P398*’. This segment docks onto the surface of ppERK2 and the N-terminus including the phosphorylatable S389’ remains disordered. Additionally, the lack of significant response to the presence of Mg^2+^/AMPPCP suggests that the small CSPs observed for *P390’* and, to a lesser extent, the α position of *S389’* (note that the β position shows no significant CSP, Fig. 2b) do not originate from the rigid positioning of the phosphorylation motif in the active site cleft but rather by non-specific collisions with the kinase surface (see below).

### Proline cis-trans isomerization has distinct effects at the F-site and the phosphorylation motif in *Elk*_387-399_

Minor states resulting from the *cis* conformations (Supplementary Fig. S4b) around the *S389’-P390’* and *F397’*-*P398’* peptide bonds (identified based on the differences in their ^13^Cβ and ^13^Cγ chemical shifts^30^; ΔCβγ_*cis*_ and ΔCβγ_*trans*_ for *P390’* are 9.3 ppm and 4.7 ppm, respectively; the corresponding values for *P398’* are 9.5 ppm and 4.7 ppm, respectively) are detectable in free *Elk*_*387-399*_. These resonances are visible not only for *S389’* and *F397’*, but also for every other position except for *S394’* and *S399’*. The relative intensities of these resonances suggest that they belong to conformers containing a single *cis*-proline.

As in case of the amide resonances corresponding to the all-*trans* form discussed above, resonances corresponding to the *cis-*containing forms at the C-terminal end of *Elk*_*387-399*_disappear at the early stages of the titration, while those at the N-terminus are only modestly perturbed and can be detected even at high ERK2 concentrations (Fig. 2a). The *S389’* and *A391’* amide resonances in the *cis*-containing forms survive throughout the titration course but display significantly smaller CSPs compared to their all-*trans* counterparts. In contrast, the *L393’, F395’*, *Q396’* and *F397’* resonances for *cis-*containing forms show small CSPs like their all-*trans* counterparts (as discussed above) and also vanish at the same stage of the titration course, suggesting very similar behavior (Fig. 2a). Additionally, the 61%-bound T_2_ values for *F395’* (80 ms) and *Q396*’ (73 ms) resonances in the *cis*-containing conformers (due to a *cis* orientation of the *F397-’P398’* peptide bond) (Fig. 3b), approach those of the residues belonging to the structured core of ppERK2 (Fig. 3d) and are significantly shorter than those of the N-terminus discussed above. These observations indicate that *cis-trans* isomerization around the *F397’-P398’* peptide bond does not have a significant effect on the F-site/FRS interactions and suggest a less critical role for the proline residue of the FxFP motif in line with previous suggestions^18^,^31^. In contrast, the relatively large CSPs for *S389’* and *A391’* amide resonances in the all-*trans* and the significantly smaller ones in the *cis* forms (Fig. 2a) suggest that isomerization around *S389’-P390’* peptide bond further compromises the already weak interaction of the N-terminal phosphorylation site with the kinase. This observation is consistent with the previously demonstrated inability of ppERK2 to phosphorylate a substrate containing a *cis-*proline immediately following the phospho-acceptor^32^.

### *Elk*_387-399_ binds ppErk2 through a diffusion-controlled process

**Figure 4:**
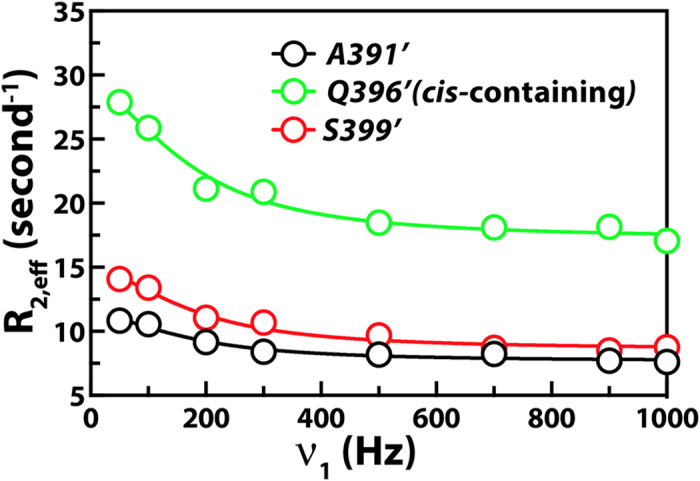
^15^N relaxation dispersion curves for selected *Elk*_*387-399*_ residues that are observable when 61% bound to ppERK2 in the presence of Mg^2+^/AMPPCP. The open circles represent experimental R_2,eff_ values and the solid lines represent fits to the fast exchange equation (Equation 4) using a global k_ex_ value. Data for *S389’*, *A391*’, *K392’*, the *cis-*containing minor state of *Q396’* (shown in green) and the C-terminal *S399’* were used in the global fit that yielded k_ex_ = 1178 ± 87 s^–1^. Note the significantly higher R_2,eff_ (∞) value for *Q396’* (*cis*) compared to those of the N-terminal residues.

We performed ^15^N relaxation dispersion experiments^33^ on *Elk*_*387-399*_ (61%-bound; the DRS was blocked using *KIM*_*15-31*_ and an excess of AMPPCP/Mg^2+^ was added to fully populate the ATP binding site such that the docked complex represents the assembled pre-catalytic state). The effective relaxation rates for the still observable residues *S389’*, *A391’*, *K392’*, *L393’*, *S399*’ belonging to the *all-*trans conformer, and the *Q396’* resonance of a *cis*-containing conformer (that has a significantly higher R_2,eff_ value indicating that it is indeed docked on to ppERK2) display a clear variation with radio-frequency field strength (Fig. 4). These dispersion curves can be fitted using a single (probability for a global fit >99.98% based on Akaike Information Criterion) global exchange rate (k_ex_ = 1178 ± 87 sec^−1^). The proline *cis*-*trans* isomerization is too slow to contribute to the k_ex_. The fact that both the N-terminal residues and the C-terminal *S399*’ displayed the same k_ex_ suggests that the observed exchange is also not due to the sampling of the active site by the N-terminus bearing the phosphorylation motif. This leads to the conclusion that this k_ex_ value characterizes the ppERK2 binding/unbinding process of *Elk*_*387-399*_. Based on the K_D,FRS_ determined above, one can estimate a k_on_ = 5.6 ± 1.0 × 10^7 ^M^−1^ sec^−1^, suggesting a largely diffusion controlled association, and a k_off_ = 451 ± 140 sec^−1^ corresponding to a lifetime of ~2 ms for the bound state. Based on the k_cat_ (0.56 ± 0.1 sec^−1^, see above) one can estimate that only 1 in about 800 docking events result in productive catalysis.

### F-site phenylalanines but not the proline make critical contacts with ppERK2 in the *Elk*_387-399_•ppERK2 complex

The interaction between *Elk*_*387-399*_ and ppERK2 is relatively weak and *ab initio* structure determination of the complex without substantial protein engineering (that is likely to perturb the interaction) is not possible. Therefore we relied on exchange-transferred ^1^H-^1^H NOEs (trNOE)^34^ to obtain structural insight into the conformation of *Elk*_*387-399*_bound to ppERK2. While ^15^N-edited (or ^13^C-edited) ^1^H-^1^H NOESY experiments on *Elk*_*387-399*_in the absence of ppERK2 suggest a less than ideal situation with the detection of a number of cross-peaks especially in the Hα-HN region, a clear increase in the number of cross-peaks and a significant increase in the intensities of existing peaks is seen upon addition of the ppERK2 (Supplementary Fig. S5 illustrates the appearance of transferred NOE cross-peaks in a ^13^C-edited 2D ^1^H-^1^H-NOESY experiment when ppERK2 is added to a NMR sample containing the *Elk*_*387-399*_peptide). Using this strategy, we were able to identify 95 unambiguous and 17 ambiguous NOEs for *Elk*_*387-399*_bound to ppERK2 from 3D ^15^N and ^13^C edited NOESY experiments. The conspicuous lack of medium range (particularly at the C-terminal region that engages ppERK2) and sequential HN-HN NOEs indicates that *Elk*_*387-399*_ adopts an extended conformation when bound to ppERK2.

In order to obtain a structural model for the *Elk*_*387-399*_•ppERK2 complex we relied on a HADDOCK-based^35^,^36^ docking protocol utilizing the experimental restraints described below. We used the crystal structure of ppERK2 (PDB ID: 2ERK)^37^ and 5 representative structures selected from the lowest energy ensemble of *Elk*_*387-399*_ (40 structures) obtained from a calculation (see Methods for details) utilizing the Aria^38^ suite and the intramolecular distance constraints described above^38^. As detailed earlier (spectral perturbations and relaxation analyses) our data clearly indicates that the N-terminal *P387’-A391*’ segment of *Elk*_*387-399*_ does not participate in any stabilizing interactions with ppERK2, therefore only the *K392’-S399’* fragment (capped by an N-terminal acetyl group) was included in the HADDOCK calculations. The experimentally determined intramolecular NOEs identified in this portion of the peptide (75 unambiguous and 16 ambiguous restraints) were included in the docking calculations. Typically, intermolecular ambiguous distance constraints involving all atoms for an interacting residue are used in HADDOCK-based docking calculations to drive the docking process. However, given the availability of spectral perturbations (CSPs and line broadening) of both backbone (as is usual) and sidechain positions for *Elk*_*387-399*_, we decided to use only those specific moieties that displayed significant perturbations (CSPs or line-broadening or appearance/disappearance) when titrated with ppERK2 (e.g. *L393’* δ methyls, the aromatic rings of *F395*’ and *F397*’, the sidechains of *Q396*’ and *K392*’) to generate ambiguous intermolecular distance restraints. For ppERK2, interacting residues were defined based using both the spectral perturbations obtained here and data from previous mutational analyses^17^,^31^. In particular, I196, M197, L198 and L235 were included based on the findings of Sheridan *et al*^31^, while the involvement of the activation loop was decided based on the NMR data presented above.

**Figure 5:**
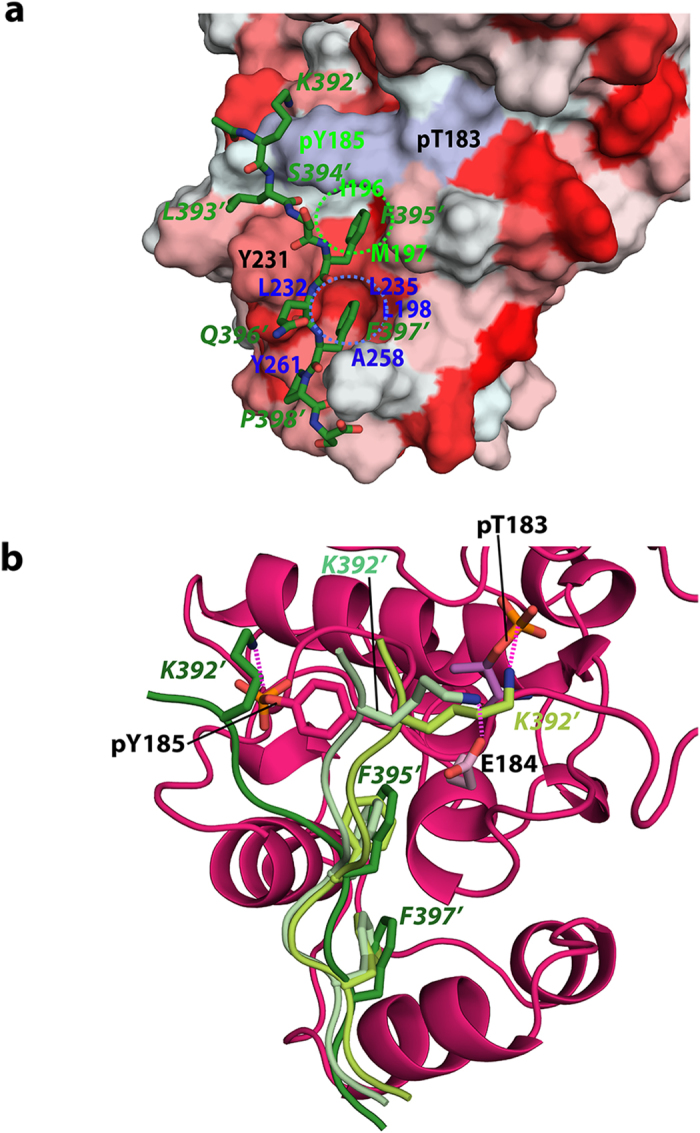
Structural features of the *ELK*_387-399•_ppERK2 complex. (**a**) The lowest energy structure of *Elk*_*387-399*_ docked onto the ppERK2 surface. The ppERK2 residues that form the two hydrophobic cavities (indicated by the dashed circles) that accommodate *F395’* and *F397’* are labeled in green and blue lettering, respectively. The ppERK2 surface has been colored using the Eisenberg generalized hydrophobicity scale^59^ (deeper red indicating more hydrophobic). The pT183 and pY185 residues are colored light sea blue. (**b**) Ribbon representation showing the best HADDOCK structure for the lowest energy cluster (*Elk*_*387-399*_is colored dark green and ppERK2 is colored pink, pY185 is shown in stick representation) compared to two structures belonging to 2^nd^ best HADDOCK cluster. For these, only the *Elk*_*387-399*_ ribbons are shown and colored pale and light green; their respective closest ppERK2 charged sidechain (E184, light blue, and pT183, dark blue) are shown as sticks. For each model, the side chains of *K392’*, *F395’*, and *F397’* on the *Elk*_*387-399*_structures are also shown as sticks. *K392’* interacts with the pT183-E184-pY185 segment (activation-lip) of the ppERK2 activation-loop in all cases. Potential hydrogen hydrogen-bonding interactions of the *K392’* sidechain with the ppERK2 activation lip are shown as dashed magenta lines: pY185 in the lowest energy structure; E184 or pT183 in the two higher energy ones. *F395’* and *F397’* show similar spatial orientations in all three cases.

The HADDOCK models are clustered within an RMSD of 2.5 Å for atoms at the *Elk*_*387-399*_•ppERK2 interface. While the lack of precise intermolecular restraints and the low density of intra-*Elk*_*387-399*_ distance restraints prevented convergence to a single unambiguous low energy configuration, all the models obtained share similar overall features. *Elk*_*387-399*_ forms an extended structure and the sidechains belonging to *F395’* and *F397’* are buried in two contiguous hydrophobic pockets (Fig. 5a). The first of these pockets is formed by I196, M197 (P+1 loop) and the aromatic ring of pY185; the second is formed by Y261, A258 (helix αL1), L232, L235 (helix αG) and L198 (P+1 loop). *L393’* is more solvent exposed, but its aliphatic side chain also interacts with Y231 (helix αG). Finally, the *K392’* sidechain interacts with the activation-loop and makes both electrostatic and hydrophobic contacts; in the models belonging to the lowest energy HADDOCK cluster, these largely involve pY185, while the charged groups of E184 and pT183 are involved in the higher energy clusters (Fig. 5b). The *P398’* ring lies at the periphery of the binding pocket, is more solvent exposed and makes a lesser number of contacts with the ppERK2 surface, in line with the limited perturbations seen in the NMR titrations. Based on these data, *F395’* and *F397’* appear to be critical for interactions of *Elk*_*387-399*_ with ppERK2, while *P398’* is relatively unimportant. The latter observation contrasts the HXMS-based model proposed by Lee *et al.*^17^, but is in line with the biochemical results of Sheridan *et al.*^31^.

## Discussion

*Elk*_*387-399*_ appears to engage ppERK2 by the insertion the F-site phenylanine aromatic rings (*F395’, F397’*) into two hydrophobic pockets with minimal involvement of the F-site proline (*P398’*). Data from a study by Sheridan *et al.*^31^ demonstrated the strict requirement for aromatic residues at the first and third positions of the F-site while replacement of the proline with phenylalanine, tryptophan or glutamine results in no appreciable loss in phosphorylation efficiency in an F-site containing substrate. We also find that while a *trans* conformation of the proline in the phosphorylation (*S389’-P390’*) motif is necessary, a *cis F397’-P398’* peptide bond does not appear to prevent binding to the FRS. The latter scenario could be physiologically relevant, given that this segment in full-length Elk-1 is predicted to be disordered and *cis* conformers could represent up to 20% of the structural ensemble. The lifetimes of these *cis*-containing states could range anywhere between several seconds to several minutes^39^, comparable to the cellular lifetimes of ppERK2 upon transient activation^40^.

While there are no high-resolution structures of complexes that illustrate F-site/FRS interactions, a crystal structure of ppERK2 bound to the anti-apoptotic protein PEA-15 that partially engages the FRS is available^41^. This small adaptor protein does not contain canonical D-site or F-site sequences and attains high-affinity in its interaction with ppERK2 by simultaneously engaging the Φ_hyd_ sub-site of the DRS and a part of the FRS. Interestingly, in the PEA-15•ppERK2 complex the hydrophobic pockets occupied by *F395’* and *F397*’ (in the complex with *Elk*_*387-399*_) are occupied by the *P73* ring and by the alkyl portion of the *R71* side-chain of PEA-15 (Supplementary Fig. S6). However a feature common to the *Elk*_*387-399*_and PEA-15 complexes is their contact with the ppERK2 activation loop; the possibility of such interactions was first raised in HXMS studies of Lee *et al.*^17^ In *Elk*_*387-399*_, this interaction is mediated by *K392’* and in PEA-15 by *R71*, despite being in different spatial orientations.

Our results also demonstrate that, upon interaction with *Elk*_*387-399*_, the activation-loop, the P+1 loop as well as the contiguous loop hosting the HRD motif become more ordered. Resonances for these regions of ppERK2, together with part of the MAPK insert also involved in binding *Elk*_*387-399*_, are not detectable in ^15^N, ^1^H TROSY spectra of ppERK2 in the absence of *Elk*_*387-399*_, suggesting the existence of significant dynamics on the μs-ms timescale in the unbound state. Methyl relaxation studies have previously identified these segments to be dynamic on the ms timescale^42^; these dynamics have been shown to be enhanced in the active form of the kinase, and are believed to involve an oscillation of the system between catalytically-competent active and inactive-like conformational states. Our results suggest that *Elk*_*387-399*_binding at the FRS stabilizes the activation loop and other portions of the catalytic machinery in what is likely an active-like conformation. Similar stabilization is not seen in the presence of D-site ligands such as *KIM*_*15-31*_.

A curious observation in our studies was the engagement of the DRS by *Elk*_*387-399*_ at high concentrations (K_D,DRS_ = 241 μM). Specifically, the spectral perturbations seen at the Φ_hyd_ sub-site for both active and inactive ERK2 are strikingly similar to those induced by *KIM*_*15-31*_. While the biological relevance of this finding is debatable, care should be taken while analyzing *in vitro* data from studies that employ F-site and D-site containing ligands simultaneously^20^,^26^. Given the significantly weaker affinity compared to a canonical D-site/DRS interaction, it is unlikely that the binding of *Elk*_*387-399*_at the DRS on its own could mediate the phosphorylation of specific Elk-1 sites *in vivo*. It is conceivable, however, that these types of weak interactions could contribute additively towards the formation of a tighter complex by engaging multiple docking sites through non-canonical interactions. Interestingly, DRS mutations result in a 2-3-fold increase in the apparent K_D_ of the interaction of ppERK2 with the F-site containing c-Fos^21^. In contrast, an Elk-1 mutant where the D-site is replaced by a second F-site exhibits only a 2.5-fold increase of the K_M_, while a 9-fold increase is observed if only the native F-site is retained^19^. PEA-15^14^,^41^ and the transcription factor Ets-1^43^,^44^ efficiently bind ERK2 despite the fact that neither protein possesses a canonical D-site or a canonical F-site. These cases provide examples of engaging multiple docking sites through non-canonical interactions that, while weak individually, lead to significantly enhanced affinity in synergistic combination.

Another major finding of this study is the independence of the F-site and the phosphorylation motifs. The spectral perturbations, and ^15^N relaxation data indicate that the *P387’-R388’-S389’-P390’* fragment is largely unrestrained and undergoes fast motions on the ps-ns timescale, populating the ppERK2 active site only transiently, while the F-site is relatively rigidly docked onto the ppERK2 FRS. This scenario suggests proximity-mediated phosphorylation^23^ involving the initial formation of a high-affinity complex with the involvement of the docking motif, followed by a second process that brings the phospho-acceptor into the catalytic site. We are unable to directly measure the insertion rate of the substrate serine (*S389’*) into the catalytic cleft. However, a lack of evidence for unique exchange processes involving *S389’* in the relaxation dispersion experiments beyond the global dynamics caused by the binding/unbinding events, suggests that this phenomenon is significantly faster than docking (and outside the dynamic regime of the CPMG-based dispersion experiments). An alternative scenario in which binding and sampling of the catalytic site take place on the same timescale appears highly unlikely since this would produce significant transient chemical shift changes (Δω in Equation 4, see Methods) for *S389’* in relaxation dispersion measurements, not seen here (data not shown).

It is now established that a proline residue following a serine/threonine phospho-acceptor is essential for efficient phosphorylation by MAP kinases^31^. A QM/MM study^45^ on ppERK2 suggests that the proline of the substrate (S/T)P motif needs to be accommodated within a hydrophobic cleft formed by L168 and the aromatic ring of pY185 to enable the optimal positioning of the phospho-acceptor within the kinase catalytic site. However, trapping of the proline within this cavity leading to the formation of a catalytically competent Michaelis-Menten complex is a relatively rare occurrence (K_eq_ ≪ 1)^44^ and the largely non-productive clashes of the proline ring with this hydrophobic cleft dominate the sampling events. We suspect that the CSPs observed for *P390’*, that are independent of the presence of Mg^2+^/AMPPCP, result from these clashes.

**Figure 6:**
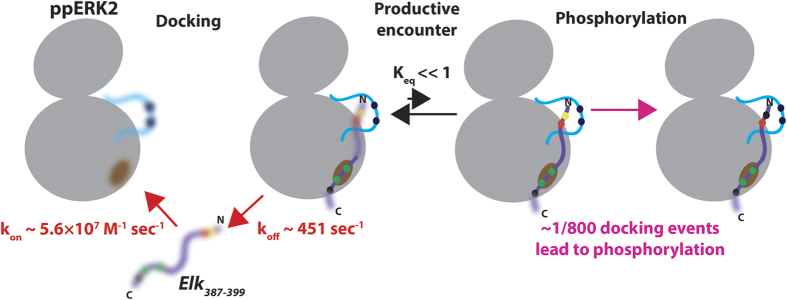
A model for FRS-driven phosphorylation of *Elk*_*387-399*_ by ppERK2. The initial diffusion-controlled docking interaction reduces the dynamics (represented by the blurred images; the blurs represent μs-ms timescale dynamics on ppERK2 and ps-ns timescale dynamics on *Elk*_*387-399*_) at the ppERK2 FRS (brown) and in the activation-loop (blue; pT183 and pY185 are represented by the dark blue spheres). The F-site phenylalanine residues (*F395’* and *F397’* green spheres) dock into two hydrophobic pockets at the FRS. The N-terminal SP motif (*S389’* represented by the yellow sphere; *P390’* represented by the red sphere) remains disordered and has largely unproductive encounters with the ppERK2 active site. The probability of these encounters (both productive as well as unproductive) is increased by the FRS/F-site docking and resultant increase in the local concentration of the phospho-acceptor near the kinase active site. A productive encounter leads to the formation of a stable Michaelis complex ultimately resulting in the phosphorylation of *S389’* (dark blue sphere).

Our results conclusively demonstrate the importance of the phenylalanine residues within F-site sequence for interactions with ppERK2 but not the F-site proline. This is demonstrated by smaller spectral perturbations, minimal stabilizing contacts with the kinase and the apparent ability of the kinase to dock conformers that contain *cis-*proline at the terminal F-site position. FRS/F-site interactions stabilize the activation and P+1 loops and a part of a MAPK insert that sample multiple conformations when the FRS is unoccupied. Our data also suggest a lack of coupling between the F-site and the phosphorylation motif with the latter remaining largely disordered even when the former is docked onto the kinase FRS. In spite of this flexibility, phosphorylation can occur due to the increased local concentration of the phosphorylation moiety and the resultant increase in the probability of a productive encounter with the kinase catalytic site (shown schematically in Fig. 6).

## Methods

### Expression and purification of ***Elk***_387-399_

Synthetic DNA corresponding to a 13-residue peptide (^387^PR***S***PAKLS**F**Q**FP**S^399^, *Elk*_*387-399*_) encompassing the F-site (in bold) and an N-terminal phosphorylation site (S389, bold italics) of Elk-1 was sub-cloned into the pTMHa vector carrying a TrpLE leader sequence^46^ between the *Hind*III and *Bam*HI restriction sites and transformed into *E. coli* BL21 (DE3) cells (*New England Biolabs*). Cells were grown in LB containing 50 mg/L of Kanamycin at 37 °C up to an OD_600_ of 0.8, pelleted down by centrifugation and washed with ice-cold M9 solution. The pellet was re-suspended in M9 medium, supplemented with ^15^NH_4_Cl (1 g/L) and uniformly-^13^C-labeled glucose (3 g/L) (*Cambridge Isotope Laboratories*) as the only sources of nitrogen and carbon, respectively. After the OD_600_ reached 0.8, protein overexpression was induced with 1 mM isopropryl-β-thiogalactopyranoside (IPTG); cells were grown for 5 hours at 37 °C under agitation. Following this, cells were harvested by centrifugation at 4000 rpm at 4 °C and lysed by sonication in a buffer containing 50 mM Tris pH 8, 1 mM EDTA, 100 mM PMSF, and 10 mg/mL of lysozyme. The resulting suspension was centrifuged at 16000 rpm for 20 minutes at 4 °C to isolate the inclusion bodies containing the TrpLE-*Elk*_*387-399*_ fusion. The resulting pellet was washed extensively with a mild detergent solution containing 50 mM Tris, 1 mM EDTA and two detergents, 1% IGEPAL CA30 and 1% deoxycholic acid (*Sigma*) followed by sonication in water and subsequent centrifugation at 18,000 rpm for 20 minutes at 4 °C. The pellet containing the inclusion bodies was then dissolved in 100% trifluoroacetic acid (TFA) by sonication. The TrpLE segment was cleaved from the fusion protein with cyanogen bromide using established strategies^47^. The cleavage reaction was performed in the dark inside Teflon sealed HPLC vials in the presence of 80% TFA. Pure ^13^C, ^15^N-labeled *Elk*_*387-399*_ peptide was obtained through reverse phase chromatography using a C18 HPLC column (*VYDAC*), and the identity of the peptide was confirmed by LC-MS. Uniformly ^15^N, ^13^C, ^2^H-labeled *Elk*_*387-399*_ was prepared in similar fashion by replacing ^13^C-labeled glucose with ^13^C, ^2^H-labeled glucose and switching to a D_2_O-based medium after adapting cells for growth in D_2_O as described previously^48^.

### Expression and purification of ppERK2

The expression and purification of ERK2 for NMR studies has been described in detail elsewhere^14^. The purified inactive protein was dialyzed into activation buffer containing 20 mM HEPES pH 7.5, 500 μM EGTA, 20 mM MgCl_2_, 2 mM DTT, and concentrated to 7 μM. The purified inactive ERK2 was activated using 0.35 μM of MKK1G7B (a constitutively active mutant of MEK1)^49^, and a total of 12 mM ATP (added at different intervals in 4 mM aliquots), and by incubating the reaction mixture at 27 °C for 8 hours. The activated protein (ppERK2, dually phosphorylated on T183 and Y185) was dialyzed into a buffer containing 20 mM Tris pH 8.0, 150 mM NaCl, 2.5 mM CaCl_2_, 0.1% β-mercaptoethanol, and purified using a MonoQ column (*GE Healthcare Biosciences*). The purified ppERK2 was then injected on a Superdex75 HiLoad 16/60 column equilibrated with the desired NMR buffer; activation was confirmed by mass-spectroscopy.

### Isothermal titration calorimetry measurements

All isothermal titration calorimetry (ITC) measurements were performed on an iTC200 microcalorimeter (*GE Healthcare Biosciences*) at 25 °C. Both *Elk*_*387-399*_ and ppERK2 were extensively dialyzed against a buffer containing 50 mM sodium phosphate pH 6.4, 150 mM NaCl, 0.5 mM EDTA, and 0.1 mM EGTA. All solutions were filtered using membrane filters of pore size 0.22 μM (*Millipore*) and degassed for about 30 minutes under vacuum. The sample cell (volume 200 μL) was filled either with a 50 μM solution of ppERK2 or buffer alone. A syringe of volume 40 μL was filled with 0.75 mM of unlabeled *Elk*_*387-399*_ (commercially synthesized and HPLC purified; *Anaspec*). Titration steps consisted of a preliminary 0.2 μL injection followed by 14 injections of 0.25 μL each. The data were fitted to a simple one-site binding model, using the ITC module embedded within the Origin 7.0 suite. The data described is representative of experiments collected in duplicate.

### NMR spectroscopy and resonance assignments

All NMR experiments were performed at 25 °C on Bruker Avance (500, 600, 700 or 800 MHz) or Varian Inova (600 MHz) spectrometers, all equipped with cryogenic probes with z-axis pulsed-field gradient capability. All spectra were processed using NMRPipe^50^ and analyzed using either Analysis (CCPN)^51^ or NMRView 52.

Typical NMR samples for assignment of ppERK2 bound to *Elk*_*387-399*_ consisted of 200–300 μM uniformly ^2^H, ^15^N, ^13^C-labeled ppERK2 with 2–3 mM unlabeled *Elk*_*387-399*_ in a buffer containing 20 mM MES pH 6.4, 150 mM NaCl, 2 mM DTT, 200 μM EDTA. The assignment strategies for ppERK2 alone and that bound to *Elk*_*387-399*_ will be described in detail elsewhere (Piserchio *et al.*, in preparation).

Samples used to obtain resonance assignments for *Elk*_*387-399*_ contained uniformly ^15^N, ^13^C-labeled *Elk*_*387-399*_ (around 300 μM) in a buffer containing 50 mM phosphate pH 6.5, 150 mM NaCl and 0.5 mM EDTA. Resonance assignments were obtained using a standard backbone-directed triple-resonance strategy^53^ using: 3D-HNCACB and 3D-CBCA(CO)NH (512, 20 and 32 complex points and sweep-widths of 10, 20 and 75 ppm in the ^1^H, ^15^N and ^13^C dimensions respectively), 3D-(H)C(CO)NH-TOCSY (512, 20 and 40 complex points and sweep-widths of 10, 20 and 53 ppm in the ^1^H, ^15^N and ^13^C dimensions respectively), and 3D-HCCH-TOCSY (512, 40 and 40 complex points and sweep-widths of 10 and 53 ppm in the ^1^H and ^13^C dimensions respectively; mixing time = 18 ms). The aromatic resonances (*F395’* and *F397’*) were assigned using the pulse sequences of Yamazaki^54^ using 512 and 28 complex points with sweep-widths of 12 and 24 ppm in the ^1^H and ^13^C dimensions, respectively.

### Titration of labeled ppERK2 with ***Elk***_387-399_ and KIM_15-31_

Spectral perturbations on ppERK2 were assessed from ^15^N, ^1^H TROSY experiments at 600 MHz and 25.0 °C using 1024 and 128 complex points and sweep-widths of 15 and 33 ppm in the ^1^H and ^15^N dimensions, respectively. A 100 μM sample of ppERK2 in a buffer containing 50 mM phosphate pH 6.8, 150 mM NaCl, 200 μM EDTA, 8 mM DTT, was titrated with (1) the following amounts of *Elk*_*387-399*_: 25.0, 49.4, 73.1, 96.2, 124.3, 177.85, 458.0, 729.9, 993.93 μM; or (2) with the following amounts of *Elk*_*387-399*_: 25.0, 49.8, 97.8, 157.7, 276.8  μM in the presence of 2.0 mM of *KIM*_*15-31*_. A 130 μM sample of ppERK2 in a buffer containing 50 mM HEPES pH 6.8, 150 mM NaCl, 200 μM EDTA and 2 mM DTT was titrated with the following amounts of *KIM*_*15-31*_: 49.2, 99,8, 149.0, 296.2, 450.6, 992.0 μM. Finally, two spectra were also collected for a 150 μM sample of inactive ERK2 alone and in the presence of 507 μM of *Elk*_*387-399*_ in phosphate buffer (described above).

### Titration of labeled ***Elk***_387-399_ with ppERK2

A reverse titration of uniformly ^15^N,^13^C-labeled *Elk*_*387-399*_with ppERK2 was performed in the following way: initially, reference spectra (see below) were collected for a sample with an equimolar amount (130 μM) of both species in a buffer containing 20 mM MES pH 6.0, 150 mM NaCl, 500 μM EDTA, 10 mM DTT, following which 140 μM of *KIM*_*15-31*_ was added; next the sample was progressively diluted with NMR buffer containing 130 μM uniformly ^15^N, ^13^C-labeled *Elk*_*387-399*_ and 140 μM of *KIM*_*15-31*_ leading to the following ppERK2 concentrations: 130, 110, 90, 60, 40, 20, 10, 5 μM. For each dilution point aliphatic ^13^C, ^1^H HSQC (15 and 50.4 ppm for the ^1^H and ^13^C dimensions, respectively), aromatic ^13^C, ^1^H HSQC (15 and 7 ppm for the ^1^H and ^13^C dimensions, respectively) and ^15^N, ^1^H HSQC (15 and 9 ppm for the ^1^H and ^15^N dimensions, respectively) experiments were collected at 25 °C at 700 MHz. The same experiments were repeated at 15° C using the following final concentrations of ppERK2: 130, 110, 60, 20, 5, 0 μM. Similar ^13^C, ^1^H and ^15^N, ^1^H HSQC experiments were also collected on a 180 μM uniformly ^15^N, ^13^C-labeled *Elk*_*387-399*_ sample containing 130 μM ppERK2 in the presence of 140 μM *KIM*_*15-31*_ in the MES buffer (described above) supplemented with 5 mM MgCl_2_ and 400 μM AMPPCP at 25.0 °C and 600 MHz.

### Effects of ***Elk***_387-399_ phosphorylation

The spectral perturbations resulting from phosphorylation of the *Elk*_*387-399*_ peptide was evaluated by adding 6.35 mM ATP to a solution containing 50 mM phosphate pH 6.5, 150 mM NaCl, 5 mM DTT, 0.5 mM EDTA, 33 μM ppERK2 and 400 μM *Elk*_*387-399*_, in the presence of 8.3 mM MgCl_2_. Chemical shift changes due to phosphorylation of *Elk*_*387-399*_ peptide were monitored using ^15^N, ^1^H HSQC experiments (4 transients; 1024 and 128 complex points for sweep-widths of 10 and 12 ppm in the ^1^H and ^15^N dimensions, respectively) and ^13^C, ^1^H HSQC experiments (1024 and 256 complex points for sweep-widths of 10 and 60 ppm in the ^1^H dimension and ^13^C dimensions, respectively).

### Analysis of spectral perturbations

Chemical shift perturbations (CSP, Δδ in ppm) induced by the ligand on uniformly ^15^N, ^13^C, ^2^H-labeled ppErk2 were calculated from the amide ^1^H (Δδ_H_) and ^15^N (Δδ_N_) chemical shift changes using:


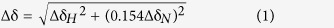


To determine substantially perturbed resonances, the following protocol derived from Schumann was used^27^. A subset of resonances with CSPs within the average plus 3-times the standard deviation (σ) was obtained through an iterative protocol where perturbations above this threshold were progressively excluded until convergence to determine the average CSP and σ values. Values above the 1σ, 1.5σ, 2σ and 3σ thresholds of the converged average were chosen to represent the degree of perturbation. Intensity ratios between corresponding resonances in the titration series were determined using normalized intensities, with the normalization factor for each spectrum defined as the average intensity calculated over a subgroup of intensities using the same protocol as the CSPs.

For uniformly ^15^N, ^13^C-labeled *Elk*_*387-399*_, the perturbations in the presence of ppERK2 were calculated using the following equation (expressed as a %):


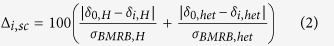


δ_0,H_ and δ_i,H_ are the chemical shifts for a particular ^1^H position (HN, Hα, Hε etc.) in free *Elk*_*387-399*_ and that at the i^th^ titration point; δ_0,het_ and δ_i,het_ are the corresponding hetero-atom shifts (N, Cα, Cε etc.); σ_BMRB,H_ and σ_BMRB,het_ are the corresponding positional standard-deviations from the BMRB.

### Analysis of binding of ***Elk***_387-399_ to the ppERK2 DRS

The affinity of *Elk*_*387-399*_ to the ppERK2 DRS was obtained by fitting the CSPs of the significantly perturbed DRS resonances with increasing concentrations of *Elk*_*387-399*_ (C_Elk_ = 49, 73, 96, 124, 178, 458, 730, 994 μM; the concentration of ppERK2, C_ppERK2_ = 93 μM) to a two-site model using the following equations^55^:


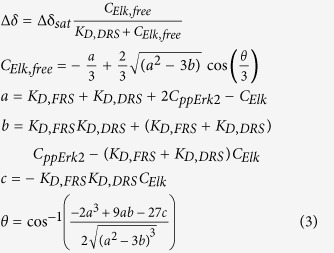


K_D,FRS_ was held constant at 8 μM (from the ITC measurements). Δδ and Δδ_sat_ are the CSPs for the DRS residues in at a given C_Elk_ and at saturation, respectively. At first, each resonance was fitted individually for Δδ and K_D,DRS_ values. This was followed by a simultaneous fit of all datasets using a global K_D,DRS_ (the Φ_chg_ residue D319 shows a complex behavior and was excluded from the global fit which used data from residues T108, L113, T116, H118, S120, D122, T158 and C159). The statistical significance of the global fit was assessed using the Akaike Information Criterion. These, and all other curve fitting described here were carried out using the GraphPad Prism 6 software suite.

### Relaxation measurements

^15^N spin-spin relaxation times (T_2_) were measured on 200 μM uniformly ^2^H, ^13^C, ^15^N-labeled ppERK2 containing 2 mM *Elk*_*387-399*_in a buffer of the following composition: 20 mM MES pH 6.4, 150 mM NaCl, 2 mM DTT and 200 μM EDTA. Data were collected at 25 °C and 600 MHz using the following relaxation delays: 0, 8.23 (2), 16.46, 24.69, 32.96 and 41.15 ms. 15 and 43 ppm spectral windows with 512 and 80 complex points was used in the ^1^H and ^15^N dimension, respectively with a 3 s recycle delay and 64 transients per increment.

^15^N spin-spin relaxation times (T_2_) were measured at 25 °C at 600 MHz on a 190 μM uniformly ^15^N, ^13^C-labeled *Elk*_*387-399*_ in the presence of 130 μM active ppERK2 and 140 μM *KIM*_*15-31*_, 5 mM MgCl_2_, 400 μM AMPPCP in a buffer containing 20 mM MES pH 6.0, 150 mM NaCl, 500 μM EDTA and 10 mM DTT. Spectral windows of 15 and 10 ppm with 512 and 32 complex points in the ^1^H and ^15^N dimension, respectively, with a 3 s recycle delay and 32 transients. The following relaxation delays were used: 10, 30, 70 (2), 110, 150, 210, 270, 350 ms. ^15^N RC-CPMG^33^ experiments (25 °C at 600 MHz) were also performed on this sample using a constant delay T_CPMG_ = 40 ms with spectral windows of 12 and 26 ppm spectral window with 512 and 24 complex points in the ^1^H and ^15^N dimensions, respectively. A 2 s recycle delay with 40 transients per increment and the following RF fields (ν_1_): 0, 50, 100, 200, 300, 500, 700, 900, 1000 Hz, were used. The CPMG data were fit to a fast two-state exchange equation (Equation 4) to obtain the exchange rate constant (k_ex_, see below) first individually and then globally. The statistical significance of the global fit was assessed using the Akaike Information Criterion.


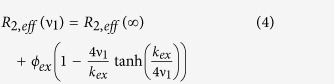


and 
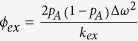


The effective relaxation rate for a particular radiofrequency field (ν_1_) is given 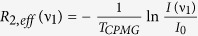 where I(ν_1_) and I_0_ are the peak intensities at the start and end of the relaxation delay (T_CPMG_), respectively. Δω represents the transient chemical shift difference; p_A_ is the population of the major state.

Finally ^15^N relaxation spin-spin relaxation rates were also collected on 130 μM *Elk*_*387-399*_ sample under identical conditions as above except in the absence of ppERK2 using the following relaxation delays: 10.0, 70.0, 170, 270.0 (2), 430.0, 590, 790 ms.

### Measurements of exchange transferred NOEs

Samples of the *Elk*_*387-399*_•ppERK2 complex for NOESY experiments were prepared by dissolving uniformly ^15^N, ^13^C-labeled *Elk*_*387-399*_ peptide (750 μM) and unlabeled ppERK2 (33 μM) in a buffer containing 50 mM phosphate pH 6.5, 150 mM NaCl and 0.5 mM EDTA. Amide and aliphatic exchange transfer NOEs (trNOE) were measured using ^15^N-edited (16 transients collected for 400, 100 and 24 complex increments over sweep widths of 10 ppm, 10 ppm and 20 ppm in the ^1^H dimensions and ^15^N dimension respectively) and ^13^C-edited (8 transients; 1024, 50 and 32 complex increments over sweep widths of 10 ppm, 10 ppm and 53 ppm in the ^1^H dimensions and ^13^C dimension respectively) 3D-NOESY-HSQC experiments with mixing times of 150 ms and recycle delays of 1.5 s. The NOESY experiment on the free *Elk*_*387-399*_ peptide was carried out under the same conditions as control.

### Determination of the structure of ***Elk***_387-399_ bound to ppERK2

To obtain an initial structural ensemble of *Elk*_*387-399*_ bound to ppERK2, trNOEs determined above were used in a Cartesian dynamics simulated annealing protocol using the Aria suite^38^. This consisted of: (1) 10000 steps of dynamics at 2000 K followed by 8000 steps of refinement; (2) 12000 steps of Cartesian dynamics while cooling from 2000 to 1000 K and then (3) 10000 steps of a second Cartesian dynamics cooling stage from 1000 to 50 K. Force constants of 10, 10 and 50 kCal mol^−1^ Å^−2^ were used for the unambiguous distance constraints during the three temperature stages of the Cartesian dynamics protocol. Prochiral Hβ and Hγ for long-chain amino acids and methyl groups from valine and leucine were treated using the floating chirality method. In all, 1024 structures were calculated of which 40 lowest energy structures (backbone RMSDs from the mean of 2.86 ± 0.71 Å) without violations were chosen to represent the structural ensemble for input into the docking protocol.

In order to perform flexible docking of the *Elk*_*387-399*_ structural ensemble onto ppERK2, only the *K392’-S399’* fragment (tr*Elk*_*387-399*_) that was deemed to be rigid upon interaction with ppERK2 was used in a HADDOCK-based docking protocol based on that described by Trellet^56^. An acetyl group was inserted at the N-terminus of the fragment to mimic the *A391’-K392’* peptide bond and to avoid the introduction of an unnatural N-terminal amine. The crystal structure of ppERK2 (PDB ID: 2ERK)^37^ and five low-energy capped tr*Elk*_*387-399*_structures representative of the ensemble were used as starting configurations. Intermolecular (ambiguous) restraints were based on spectral perturbations and mutation data (see Results section) and intramolecular restrains constituted of the distances obtained from the trNOE experiments. For each of the five initial tr*Elk*_*387-399*_conformations, 4000 docked models were created through a rigid body minimization protocol for a total of 20000 complexes, then the top scoring 600 structures underwent a semi-flexible simulated annealing protocol consisting of the following steps: (1) 2000 steps (2 fs) of rigid body dynamics at 2000 K and 2000 cooling steps to 500 K; (2) 4000 steps of a semi-flexible simulated annealing protocol in torsion angle space starting at 500 K and ending at 50 K, where the side chains at the protein/peptide interface were allowed to move; (3) finally 4000 steps of semi-flexible simulated annealing starting at 500 K and ending at 50 K where both backbone and sidechains at the complex interface were allowed to move. During these three stages all tr*Elk*_*387-399*_and ppERK2 atoms belonging to the activation-loop residues D173 through R189 were fully flexible. The interface was defined as those atoms from the two species within 5 Å of each other. Finally, all the resulting structures underwent refinement in explicit solvent consisting of 100 heating steps at 100 K, 200 K and 300 K followed by 1250 sampling steps at 300 K and 2000 cooling steps at 300 K, 200 K and 100 K.

### Kinase assays

Kinase assays to determine the Michelis-Menten parameters for the phosphorylation of *Elk*_*387-399*_by ppERK2 were performed in 100 μL volumes at 30 °C in a buffer containing 25 mM HEPES pH 7.5, 50 mM KCl, 0.1 mM EDTA, 0.1 mM EGTA, 2 mM DTT, 10 mM MgCl_2_, and 40 μg/mL BSA. Reaction mixtures contained 2 nM ppERK2, and 1.6–400 μM *Elk*_*387-399*_. The reaction mixture was incubated for 10 minutes at 30 °C before initiation with 500 μM γ-^32^P ATP (specific activity of 1 × 10^15 ^cpm/mol). Aliquots (10 μL) were spotted onto P81 filter papers at fixed times. The filter papers were washed three times for 15 minutes with 50 mM phosphoric acid to remove excess ATP and washed once with acetone for drying. The amount of phosphate incorporated into *Elk*_*387-399*_was determined by the associated counts per minute on a scintillation counter (*Packard 1500*) at a σ value of 2.

Fluorescence-based kinase assays were performed at 28 °C in a buffer containing 25 mM HEPES, pH 7.5, 50 mM KCl, 2 mM DTT, 0.1 mM EDTA, 0.1 mM EGTA, 0.5% glycerol, 2.5 mg/mL BSA, 10 mM MgCl_2_, 2 nM ppERK2 with various concentrations of the ERK-sensor-D1 peptide in the absence or the presence of 100 μM *Elk*_*387-399*_. The ERK-sensor-D1 peptide^57^ (QRKTLQ**RR**NLKGLN**L**N**L**-X_3_-TGPL***S***PC-Sox-PF) is a highly efficient ERK2 substrate and includes – (1) a D-site sequence (bold) derived from the yeast MAP kinase kinase (MKK) Ste7, (2) a phosphorylatable serine (bold italics) fused to the C-terminus using a flexible linker (X = 6-aminohexanoic acid) and (3) the cysteine at the +2 position modified by a sulfonamido-oxine (Sox) group. The reaction was initiated after 1 hour of pre-incubation at room temperature by adding 10 μL of 5 mM Mg^2+^/ATP to 90 μL of assay mixture and the fluorescence intensity was recorded at each time point for a total of 6 minutes on a Fluorolog fluorimeter (*Horiba Jobin Yvon*) in quartz cuvettes (*Starna Cells*) with a 10 mm path length and a reaction volume of 100 μL. The sample was excited at 360 nm and fluorescence was monitored at 492 nm using excitation and emission slit widths of 3 nm. The change in fluorescence was related to the progression of the reaction using a previously determined conversion factor^58^.

## Additional Information

**How to cite this article**: Piserchio, A. *et al.* Structural and Dynamic Features of F-recruitment Site Driven Substrate Phosphorylation by ERK2. *Sci. Rep.* **5**, 11127; doi: 10.1038/srep11127 (2015).

## Supplementary Material

Supplementary Information
