# Supplementary material for: Structural and Dynamic Features of F-recruitment Site Driven Substrate Phosphorylation by ERK2
Source: Sci Rep. 2015 Jun 8;5:11127. doi: 10.1038/srep11127 (PMC4459106; doi:10.1038/srep11127)
Supplement: Supplementary Information [file srep11127-s1.pdf]

SUPPORTING INFORMATION FOR

Structural and Dynamic Features of F-recruitment Site Driven Substrate Phosphorylation by ERK2

Andrea Piserchio, Venkatesh Ramakrishan, Hsin Wang, Tamer S. Kaoud, Boris Arshava, Kaushik Dutta, Kevin N. Dalby, and Ranajeet Ghose

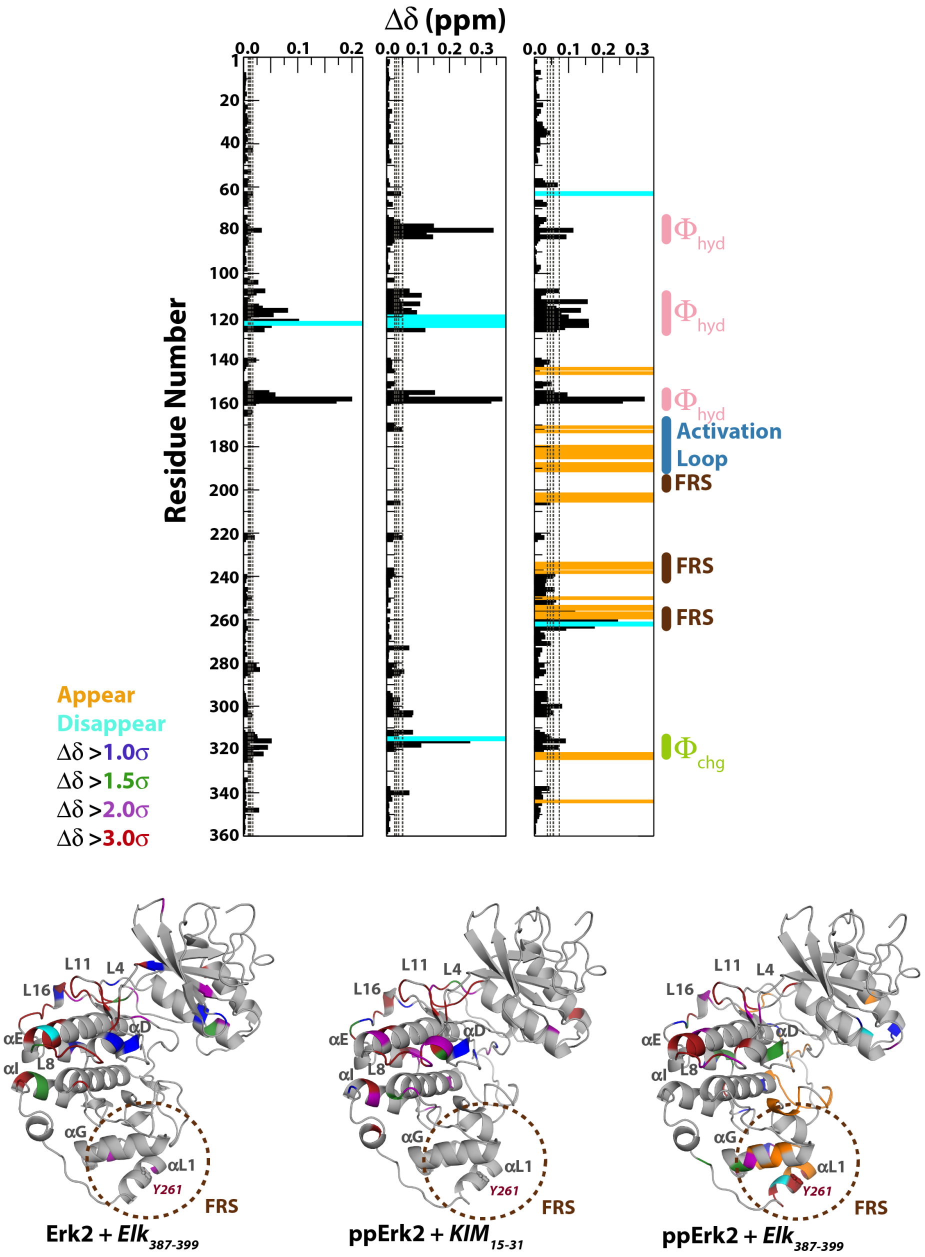

Figure S1. Spectral perturbations induced on inactive ERK2 by *Elk*$_{387\text{-}399}$ at an ERK2: *Elk*$_{387\text{-}399}$ ratio of 1:3.38 (top and bottom left panels), on ppERK2 by *KIM*$_{15\text{-}31}$ (ratio 1:9.9, middle panels) or by *Elk*$_{387\text{-}399}$ (ratio 1:9.9, right panels). Residues for which the corresponding resonances are absent in the ligand-free state but appear in its presence are shown in orange; residues for which the resonances disappear in the presence of ligand are shown in cyan. The position of the critical Y261 residue is labeled. Chemical shift perturbations (CSPs) have been calculated using Equation 1. The corresponding perturbations are mapped onto the crystal structure of inactive (left) or ppERK2 (middle, right). CSPs that are 1.0σ, 1.5σ, 2.0σ or 3.0σ above the mean (see Methods) are colored blue, green, purple and red respectively. Key secondary structure elements that form the DRS and the FRS and are discussed in the main text are labeled in grey.

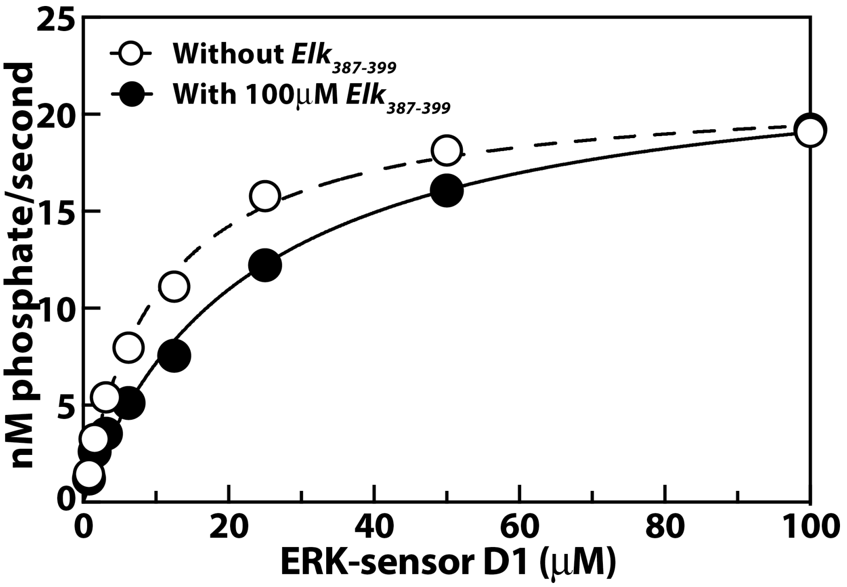

Figure S2. Activity of ppERK2 (2 nM) towards the Erk-sensor-D1 peptide substrate. This peptide (QRKTLQ**RR**NLKGLN**L**N**L**-X_3-TGPL***S***PC-Sox-PF) is a highly efficient ERK2 substrate and includes a D-site sequence (bold) derived from the yeast MAP kinase kinase (MKK) Ste7, a phosphorylatable serine (bold italics) fused to the C-terminus by a flexible linker (X=6-aminohexanoic acid) and a sulfonamido-oxine (Sox) group modified cysteine at the +2 position. Activity was measured in the absence (open circles, experimental data; dashed line, fits to the Michelis-Menten equation) and presence (filled circles, solid line; 100 μM $Elk_{387-399}$) of $Elk_{387-399}$. The K_M increases from 10.1±0.8 μM to 22.7±2.8 μM without any significant changes in V_{max} (21.4±0.5 and 23.4±1.1 nM phosphate/second). At this concentration, the occupancy at the kinase DRS is approximately 29% (based on the $K_{D,FRS}$ of 8 μM and a $K_{D,DRS}$ of 242 μM).

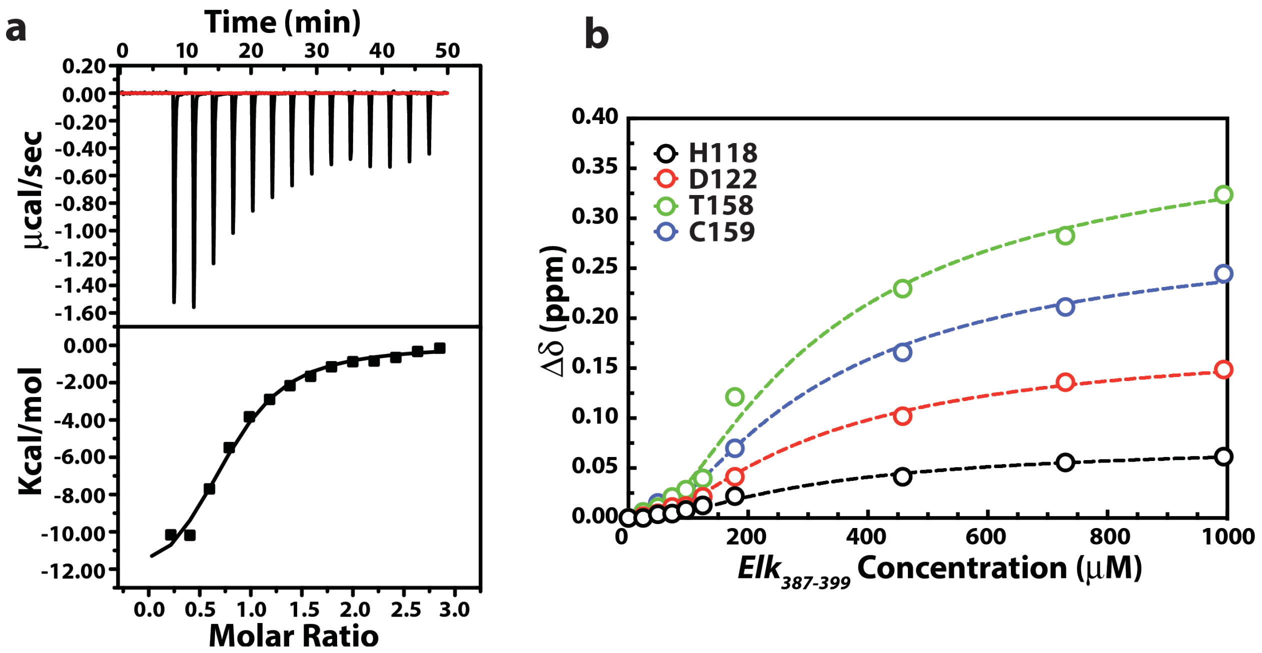

Figure S3. (a) Isothermal calorimetry (ITC) measurements on *Elk*$_{387-399}$. The ppERK2 concentration was held constant at 50 μM. The data were fit to a one-site binding model and yielded a $K_{D,FRS}$ of 8±0.9 μM. This approach provides an accurate measure of $K_{D,FRS}$ since the occupancy of the DRS is expected to be ~6% at an equimolar ratio of ppERK2 to *Elk*$_{387-399}$. **(b)** An estimate of $K_{D,DRS}$ was obtained by fitting the chemical shift changes at DRS in the presence of increasing concentration of *Elk*$_{387-399}$ at a constant ppERK2 concentration to a two-site model (Equation 3). The $K_{D,FRS}$ was held constant at 8 μM in fitting the chemical shift changes for residues T108, L113, T116, H118, S120, D122, T158 and C159 to Equation 3. A statistically significant global $K_{D,DRS}$=242.0±15.5 μM was obtained. Experimental data for a selected subset of residues is shown, along with the fitted curves (dashed lines), assuming a global $K_{D,DRS}$. The large difference in $K_{D,FRS}$ and $K_{D,DRS}$ leads to the sigmoidal binding curves characteristic of sequential two-site binding.

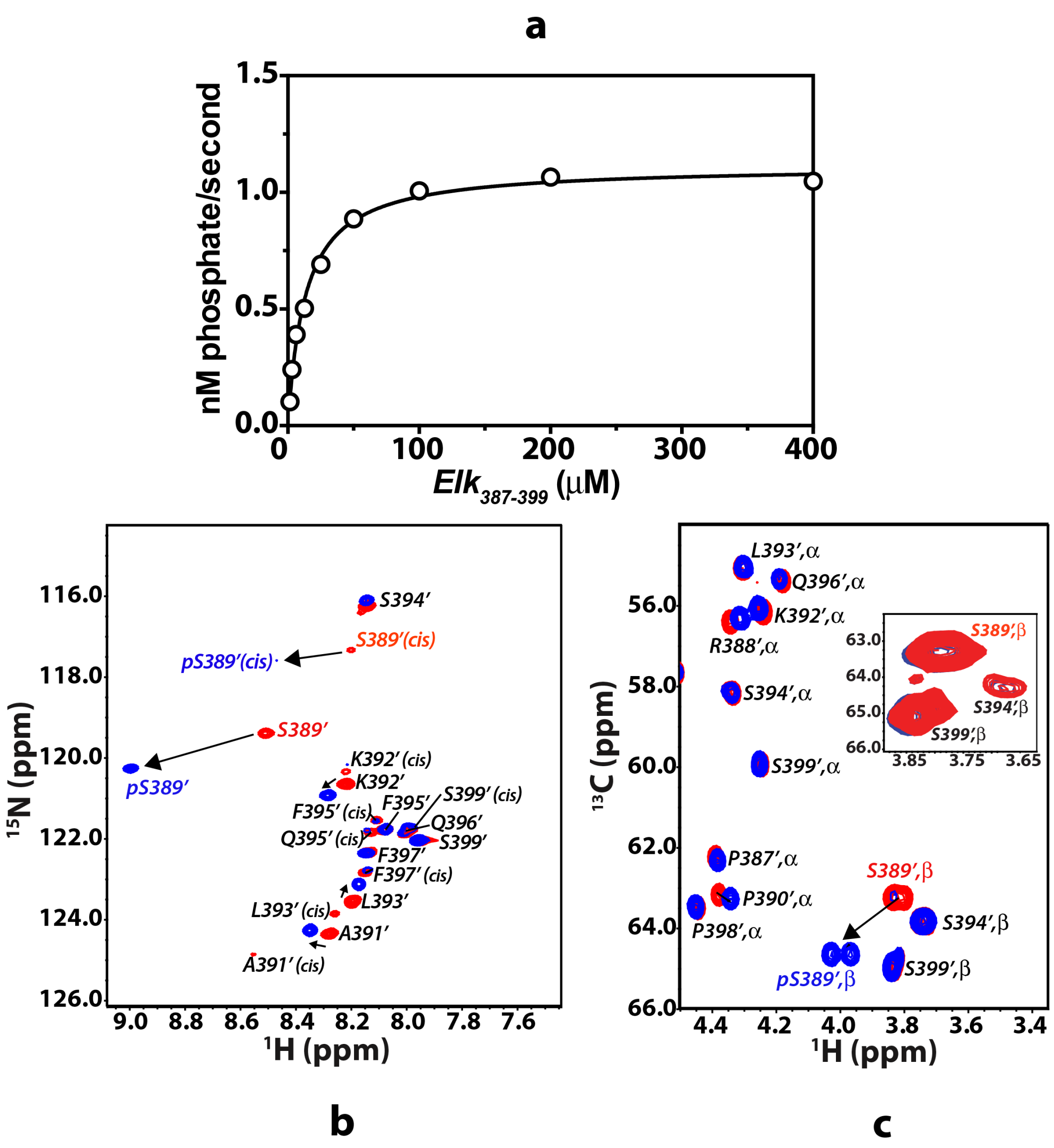

Figure S4. (a) Phosphorylation of *Elk387-399* by ppERK2 measured through the incorporation of radiolabeled phosphate from γ-^{32}P-ATP. The open circles represent the experimental incorporation at different concentrations of substrate *Elk387-399*. The solid line represents a fit to the Michaelis-Menten equation yielding k_{cat}=0.56±0.1 sec^{-1} and K_M=13.4±1.1 µM. ^{15}N,^{1}H HSQC **(b)** and ^{13}C,^{1}H HSQC (showing an expansion of the α/β region) **(c)** spectra of a 400 µM sample *Elk387-399* in the presence of a 33 µM ppERK2 in a buffer containing 50 mM phosphate pH 6.5, 150 mM NaCl, 5 mM DTT, 0.5 mM EDTA and 6.5 mM ATP, in the absence (red) or the presence of 8.3 mM Mg^{2+} (blue). The phosphorylation reaction is completed in the presence of Mg^{2+}. Notably, no significant perturbations are noted when ATP is replaced by AMPPCP in the presence of Mg^{2+} (inset). *S389'* is labeled in red and blue in the dephosphorylated and phosphorylated states, respectively. The resonance corresponding to the *cis*-containing conformer of *pS389'* has been tentatively assigned. New positions of selected resonances that show large chemical shift changes upon phosphorylation are indicated by the arrows.

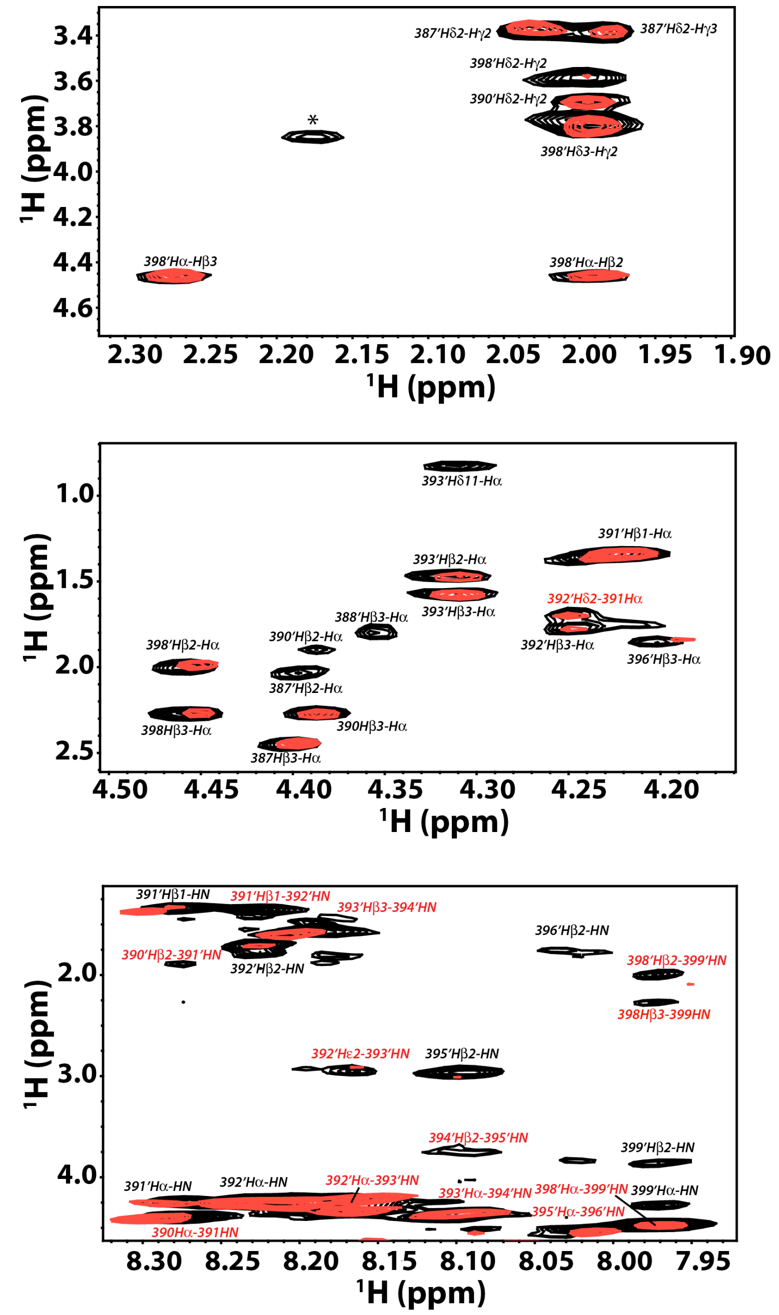

Figure S5. Expansions of selected regions of a 2-dimensional ^{13}C-edited ^{1}H, ^{1}H NOESY spectra (150 ms mixing time; 600 MHz) of a 0.75 mM sample of $Elk_{387\text{-}399}$ in the absence (red) or the presence of 0.033 mM ppERK2 (23:1 $Elk_{387\text{-}399}$:ppERK2 molar ratio; black). The appearance of exchange transferred NOE cross-peaks in the presence of ppERK2 is evident. Peaks has been labeled using IUPAC nomenclature as ω1, ω2 indicating the indirect, direct dimensions, respectively. An asterisk (*) indicates a NOE originated by a *cis* isomer and that has not been used for structure calculation. The intra-residue NOEs have been labeled in black and the sequential NOEs have been labeled in red.

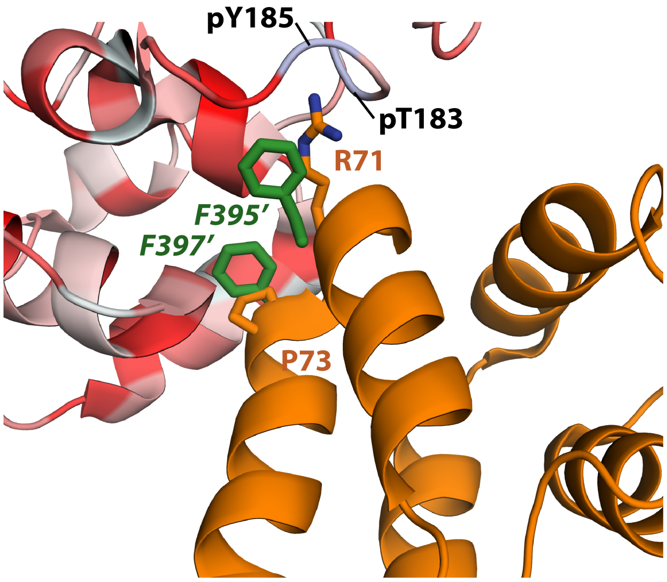

Figure S6. The spatial positions occupied by the critical *Elk*$_{387\text{-}399}$ F-site residues *F395'* and *F397'* (shown in stick representation and colored green) in the *Elk*$_{387\text{-}399}$•ppERK2 complex are occupied by the alkyl portion of the R71 sidechain and P73, respectively, in the PEA-15•ppERK2 complex (PEA-15 is shown in ribbon representation and colored orange; R71 and P73 are shown in stick representation). Note that PEA-15 contains neither a canonical D-site nor a canonical F-site sequence, but partially engages both the DRS and the FRS on ppERK2. The ppERK2 ribbon has been colored using the Eisenberg hydrophobicity scale (deeper red indicates more hydrophobic); pT183 and pY183 have been colored light sea blue and labeled.
